# Supplementary material for: Perilla frutescens Leaf Alters the Rumen Microbial Community of Lactating Dairy Cows
Source: Microorganisms. 2019 Nov 13;7(11):562. doi: 10.3390/microorganisms7110562 (PMC6921060; doi:10.3390/microorganisms7110562)
Supplement: Supplementary file 1 [file microorganisms-07-00562-s001.pdf]

Table S1. Ingredients and chemical composition of the diets

Item	Diet
Ingredients, g/kg of DM	
Corn grain	141.3
Steam-flaked corn	101.2
Bran	7.40
Soybean meal	94.6
Extruded Soybean	31.2
Rapeseed meal	23.1
Sugar beet meal	64.4
DDGS	23.4
Corn silage	243.4
Alfalfa hay	160.2
Oat hay	47.4
Premix concentrate[a]	62.3
Chemical composition, g/kg of DM unless noted	
Organic matter	927.1
Crude protein	174.0
Neutral detergent fiber	284.2
Acid detergent fiber	156.4
Ether extracts	21.8
NE_L[b], Mcal/kg of DM	1.70

[a]Formulated to contain (per kilogram of premix concentrate) 365 g of soybean meal; 182.5 g of fat powder; 73 g of $CaHCO_3$; 146 g of $NaHCO_3$; 92 g of stone powder; 87.6 g of NaCl; 36.5 g of MgO_2; 30 mg of Se; 15 mg of Co; 385 mg of Cu; 61 mg of Fe; 1065.5 mg of Mn; 58 mg of I; 2459 mg of Zn; 730,000 IU of vitamin A; 1750,000 IU of vitamin D; 458 IU of vitamin E.

[b]Calculated based on Ministry of Agriculture of P. R. China recommendations [1].

Table S2. The relative proportion of bioactive compounds in *Perilla frutescens* leaf (PFL) using UHPLC-QTOF-MS

Bioactive compounds	Proportion, %
Clareolide	13.10
Betaine	8.26
Sucrose	7.48
Scutellarin	6.15
Apigenin	5.24
L-Valine	4.79
Caffeic acid	4.69
2-Pyrrolidinecarboxylic acid	3.56
L-Phenylalanine	2.88
L-Tryptophan	2.61
Adenosine	2.38
Luteolin 7-glucuronide	2.25
5-Acetylsalicylic acid	2.20
Citric acid	2.03
L-Leucine	1.85
Stachydrine	1.61
Apigenin 7-O-glucuronide	1.54
Oroxylin A	1.48
Adenine	1.40
7-Hydroxycoumarin	1.24
Esculetin	1.11
Luteolin	1.04
Orsellinic acid	0.94
Cynaroside	0.87
Chrysin	0.71
Vicenin II	0.66
Kaempferol-7-O-β-D-glucopyranoside	0.59

Gossypol-acetic acid	0.59
Baicalin	0.58
L-Tyrosine	0.57
L(-)-Carnitine	0.48
Manninotriose	0.48
α-Cyperone	0.47
Nicotinic acid	0.46
p-Hydroxybenzaldehyde	0.46
Rosmarinic acid	0.44
Nicotinamide	0.41
Dihydroartemisinic acid	0.41
Kaempferol	0.39
Isovitexin	0.39
Azelaic acid	0.39
Hydroxygenkwanin	0.38
Uridine	0.38
Salicylic acid	0.37
Protocatechualdehyde	0.36
Senkyunolide	0.35
Danshensu	0.32
5-Hydroxymethylfurfural	0.30
Gentisic acid	0.28
Maleic acid	0.27
Guanosine	0.25
Pinocembrin	0.25
Glabrolide	0.24
L-Glutamic acid	0.23
18 β-Glycyrrhetintic Acid	0.22
Scutellarein	0.21

β-Elemonic acid	0.21
Ligustilide	0.21
3-n-Butylphathlide	0.20
Calcium pantothenate	0.20
Oleanonic acid	0.19
6-Shogaol	0.18
Quillaic acid	0.18
Hispidulin	0.18
Vitexin	0.18
3,5-Dimethoxy-4-hydroxybenzaldehyde	0.17
Curcumenol	0.16
Germacrone	0.15
Perillene	0.15
α-Linolenic acid	0.15
Ursonic acid	0.15
1-Caffeoylquinic acid	0.14
Camphor	0.14
Pectolinarigenin	0.14
(+)-Nootkatone	0.13
4-Hydroxybenzoic acid	0.13
Quercetin 3-O-β-D-Glucuronide	0.13
Ursolic acid	0.13
Cytidine	0.12
Astragalin	0.12
p-Hydroxy-cinnamic acid	0.12
Methyl rosmarinate	0.11
Diosmetin	0.11
Propylparaben	0.10
Vanillin	0.10

6-Gingerol	0.10
Isoguanosine	0.09
Curcumol	0.09
Abscisic acid	0.08
7,8-Dihydroxycoumarin	0.08
Sec-O-Glucosylhamaudol	0.08
Aurantio-obtusin	0.08
Artemisinic acid	0.07
Formononetin	0.07
3,4-Dihydroxyphenylethanol	0.07
Genkwanin	0.07
Amygdalin	0.07
2-Adamantanone	0.07
Ferulic acid	0.07
Arglabin	0.05
Sinapic acid	0.05
Scoparone	0.05
Protocatechuic acid	0.05
Pinoresinol 4-O-glucoside	0.05
Dehydroandrographolide	0.04
Genistein	0.04
Isoquercitrin	0.04
Atractylodin	0.04
Phenethyl caffeate	0.04
p-Coumaric acid	0.04
Naringenin chalcone	0.04
Lupenone	0.04
Quinic acid	0.04
Asiatic acid	0.04

Isoferulic acid	0.03
Ethyl caffeate	0.03
Citropten	0.03
Artemisinin	0.03
Lovastatin	0.03
Guanine	0.03
(+)-Pinoresinol	0.03
Eriodictyol	0.02

Reference

1. MOA (Ministry of Agriculture of P.R. China). 2004. Feeding Standard of Dairy Cattle (NY/T 34–2004). MOA, Beijing, China.
